# Endoscopic submucosal dissection of early gastric cancer with mixed-type histology: Protocol for a systematic review and meta-analysis

**DOI:** 10.1097/MD.0000000000013838

**Published:** 2018-12-21

**Authors:** Chang Seok Bang, Jae Ho Choi, Young Joo Yang, Jae Jun Lee, Gwang Ho Baik

**Affiliations:** a Department of Internal Medicine; b Institute of New Frontier Research; c Department of Anesthesiology and Pain Medicine, Hallym University College of Medicine, Chuncheon, Korea.

**Keywords:** early gastric cancer, endoscopic submucosal dissection, histologic heterogeneity, mixed-type histology

## Abstract

**Background:**

Endoscopic submucosal dissection (ESD) is a primary treatment for the early gastric cancer (EGC) who have a negligible risk of lymph node metastasis satisfying specific criteria. These criteria are histologically categorized by EGC with differentiated-type histology (EGC-DH) and undifferentiated-type histology (EGC-UH). However, gastric cancer is histologically heterogenous and there has been no specific criteria for EGC with mixed-type histology (EGC-MH). Moreover, therapeutic outcomes of ESD for EGC-MH have not been clearly described.

**Methods:**

We will search the core databases (MEDLINE (through PubMed), the Cochrane Library, and Embase) from their inception to November 2018 using pre-established searching strategy by 2 independent evaluators. The P.I.C.O. is as follows; Patients: who have EGC-MH, Intervention: ESD, Comparison: none, Outcome: at least one among the rate of complete resection, curative resection, en bloc resection, recurrence or procedure-related adverse event that enabled an evaluation of feasibility of ESD. All types of study design will be sought and publications in English with full-text will be included. The risk of bias will be assessed using the ROBINS-I tool. Descriptive data synthesis is planned and quantitative synthesis will be used if the included studies are sufficiently homogenous (pooled therapeutic outcomes data with 95% confidence intervals). Publication bias will be assessed with quantitative analyses if more than 10 articles are enrolled.

**Results:**

The results will provide evidence for validity of current ESD criteria in addition to the technical feasibility of ESD for EGC-MH.

**Conclusion:**

This study will provide evidence of ESD for EGC-MH.

## 1 Introduction

Endoscopic submucosal dissection (ESD) is a primary treatment for early gastric cancer (EGC) who has a negligible risk of lymph node metastasis (LNM).^[1]^ This enables an en bloc resection of the EGC and stomach preservation, thereby avoiding invasive surgery. After histologic confirmation (or even without pre-ESD histologic confirmation), ESD of a lesion satisfying the specific indication is performed. Pathologic confirmation of the resected specimen after ESD determines whether curative resection was achieved (satisfying ESD criteria), which implies a favorable long-term outcome. These indication or criteria are histologically categorized by EGC with differentiated-type histology (EGC-DH) and undifferentiated-type histology (EGC-UH).^[1,2]^

The absolute indications for ESD of EGC include EGC-DH of less than 2 cm in the absence of ulceration and lymphovascular invasion (LVI).^[2]^ The indications for ESD have been expanded with advances in endoscopic skills and expertise and these include mucosal EGC-DH without ulceration irrespective of tumor size; mucosal EGC-DH with ulceration measuring less than 3 cm; mucosal EGC-UH measuring less than 2 cm without ulceration; EGC-DH with minute submucosal invasion (≤500 μm, SM1) measuring less than 3 cm;, without evidence of LVI.^[3–5]^

Currently, differentiated-type predominant EGC mixed with an undifferentiated component is considered as EGC-DH, whereas undifferentiated-type predominant EGC mixed with a differentiated component is considered as EGC-UH.^[6]^ However, gastric cancer is histologically heterogenous and diagnosis of EGC with mixed-histology (EGC-MH; EGC with histologic heterogeneity) is challenging. It is only diagnosed after ESD or surgery, as pure-type gastric cancer and initial biopsy can be changed after therapeutic resection.^[7]^ There have been no specific criteria of ESD for EGC with mixed-type histology (EGC-MH). Moreover, therapeutic outcomes of ESD for EGC-MH have not been clearly described. The aim of this study is to evaluate the efficacy and safety of ESD for EGC-MH.

## 2 Methods

This systematic review and meta-analysis will fully adhere to the principles of the Preferred Reporting Items for Systematic reviews and Meta-Analyses (PRISMA-P) checklist.^[8]^ This study was registered at PROSPERO (https://www.crd.york.ac.uk/prospero) on November 2018 (registration number, CRD42018114283) before study was initiated. The approval of institutional review board was exempted due to the characteristics of this study (collecting and synthesizing data from published studies).

### 2.1 Literature searching strategy

MEDLINE (through PubMed), the Cochrane library, and Embase will be searched using common keywords associated with ESD for EGC-MH (from inception to November 2018) by 2 independent evaluators (C.S.B., and J.H.C). Medical Subject Heading or Emtree keywords will be selected for searching electronic databases. The abstracts of all identified studies will be reviewed to exclude irrelevant publications. Full-text reviews will be performed to determine whether the inclusion criteria are satisfied in the remaining studies, and the bibliographies of relevant articles will be rigorously reviewed to identify additional studies. Disagreements between the evaluators will be resolved by discussion or consultation with a third evaluator (K.H.B.). The detailed searching strategy is described in Table 1.

**Table 1:**
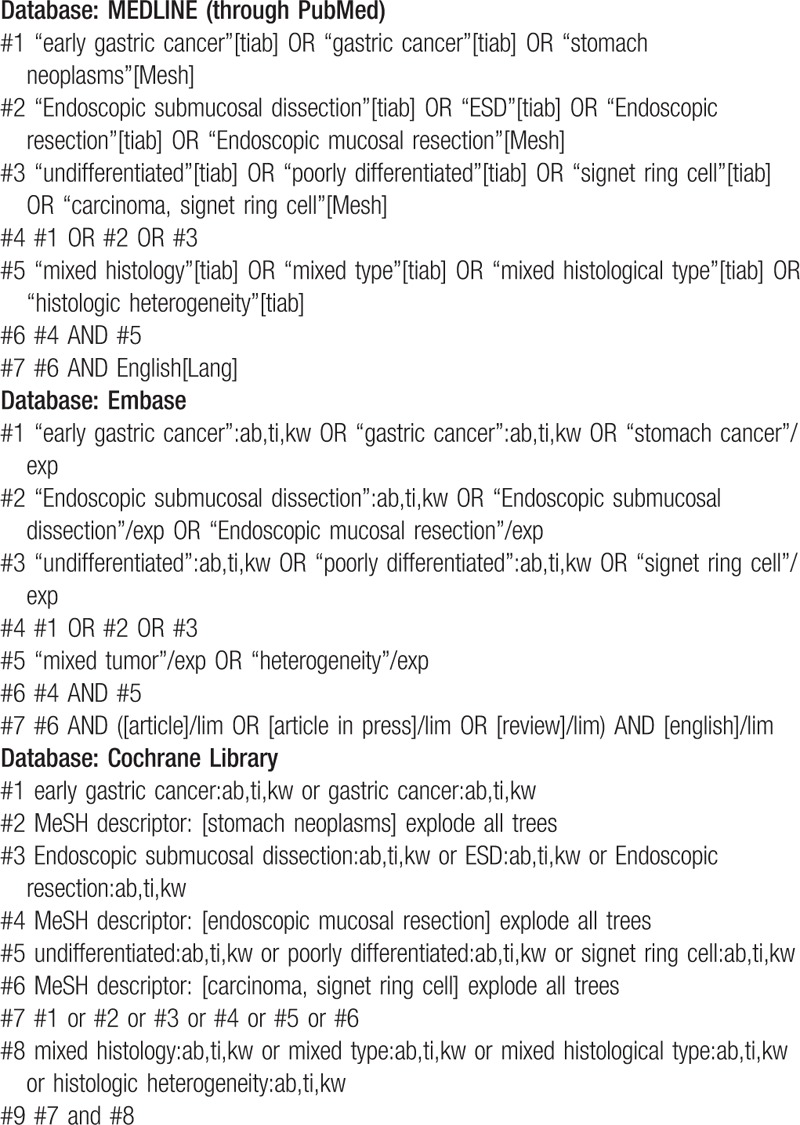
Searching strategy to find the relevant articles.

### 2.2 Selection criteria

We will include studies that met the following criteria: patients: who have EGC-MH (histologic heterogeneity); intervention: ESD or other types of endoscopic resection (i.e., endoscopic mucosal resection); comparison: none; outcome: at least one among the rate of complete resection, curative resection, en bloc resection, recurrence, or procedure-related adverse event that enabled an evaluation of feasibility of ESD; study design: all types, including randomized, prospective, or retrospective studies; studies of human subjects; publications in English; and full-text publications. Studies that met all of the inclusion criteria will be sought and selected. The exclusion criteria are as follows: review articles; guidelines, consensus documents, or expert position papers; comments, letters, brief reports, proceedings, or protocol studies; case reports; publications with incomplete data; and meta-analysis articles. Studies meeting at least 1 of the exclusion criteria will be excluded from this analysis.

### 2.3 Methodological quality

The methodological quality of the included publications will be assessed using the Risk Of Bias In Non-randomized Studies - of Interventions (ROBINS-I, formerly named as Cochrane Risk of Bias Assessment Tool: for Non-Randomized Studies of Interventions) tool.^[9]^ The ROBINS-I tool contains 7 domains, including “bias due to confounding” and “bias in selection of participants into the study” at pre-intervention, “bias in classification of intervention” at intervention and “bias due to deviations from intended interventions,” “bias due to missing data,” “bias in measurement outcomes,” and “bias in selection of the reported result” at postintervention.^[9]^ Each domain is determined to exhibit low, moderate, serious, or critical risk of bias. No information category will be used only when insufficient data are reported to permit a judgment.^[9]^ Overall risk of bias judgment is determined based on the interpretation of each domain level and low risk indicates that the study is comparable to a well-performed randomized trial for all domains being evaluated. Moderate risk of bias indicates the evidence of study is sound for a nonrandomized study but not comparable to a randomized trial (low or moderate risk of bias for all domains). Serious risk of bias indicates the presence of important problems (serious risk of bias in at least one domain, but not at critical risk of bias in any domain). Critical risk of bias indicates the study is problematic to provide any useful evidence (critical risk of bias in at least one domain).^[9]^

Two of the evaluators (C.S.B. and J.H.C.) will independently assess the methodological qualities of all the included studies, and any disagreements between the evaluators will be resolved by discussion or consultation with a third evaluator (G.H.B.).

### 2.4 Primary and modifier-based analyses

Two evaluators (C.S.B. and J.H.C.) will independently use the same data fill-in form to collect the primary summary outcome and modifiers in each study, and disagreements between the 2 evaluators will be resolved by discussion or consultation with a third author (G.H.B). The definition of primary therapeutic outcome is as follows; En bloc resection is defined as complete removal of cancer in a single piece without fragmentation. Complete resection is defined as removal of cancer with no neoplastic components at the lateral or vertical margins and without LVI on microscopic examination. Curative resection is defined as removal of cancer with 20 mm or smaller intramucosal lesions without ulceration (scar), neoplastic components at the lateral or vertical margins, and LVI for EGC-UH; removal of cancer with 20 mm or smaller intramucosal lesions without ulceration; removal of cancer with 20 mm or larger intramucosal lesion without ulceration (scar); removal of cancer with less than 30 mm intramucosal lesion with ulceration (scar); and removal of cancer with less than 30 mm submucosal invasion depth of <500 μm, without ulceration (scar), neoplastic components at the lateral or vertical margins, and LVI for EGC-DH.^[1,2,4]^ If the lesion does not meet these curative criteria, it is regarded as noncurative resection.^[1,2,4]^ Recurrence is defined as reappeared at the site of the lesion (local recurrence) or synchronous, metachronous, or distant metastatic lesions, and adverse event of ESD is defined as the cancers whose treatment resulted in procedure-related gastric hemorrhage or perforation.^[3]^

Narrative (descriptive) synthesis is planned and quantitative synthesis will be used if the included studies are sufficiently homogenous. Authors previously reported therapeutic outcomes of ESD for EGC-UH using pooled meta-analysis of crude outcomes of each study.^[10]^ The common effect size will be extracted from each study using method previously described (pooled meta-analysis of crude outcomes)^[10]^ and we will also perform sensitivity analyses and meta-regression using the modifiers identified during the systematic review to confirm the robustness of the main result and to identify the reason of heterogeneity.

### 2.5 Statistical analysis

Comprehensive Meta-Analysis Software (version 3, Biostat; Borenstein M, Hedges L, Higgins J, and Englewood RH, NJ) and R version 3.2.3^[11]^ will be used for this meta-analysis. We will calculate the pooled rate of an en bloc resection, complete resection, curative resection, recurrence and adverse event rates divided by gastric hemorrhage and perforation, whenever possible. Heterogeneity will be determined using the *I*^*2*^ test developed by Higgins, which measures the percentage of total variation across studies.^[12]^*I*^*2*^ will be calculated as follows: *I*^*2*^ (%) = 100 × (Q-df) / Q, where Q is Cochrane heterogeneity statistic, and df signifies the degrees of freedom. Negative values for *I*^*2*^ will be set to zero, and an *I*^*2*^ value over 50% was considered to be of substantial heterogeneity (range: 0–100%).^[13]^ Pooled-effect sizes with 95% confidence intervals (95% CIs) will be calculated using the DerSimonian and Laird random effects model meta-analysis and sensitivity analyses will be performed using the Mantel--Haenszel fixed-effect model meta-analysis.^[14]^ These results will be confirmed by the *I*^*2*^ test. Significance will be set at *P* = .05. Publication bias will be evaluated using Begg funnel plot, Egger test of the intercept, Duval and Tweedie trim and fill, and Begg and Mazumdar rank correlation test.^[15–19]^

## 3 Discussion

This is the protocol of a systematic review and meta-analysis for the therapeutic outcomes of ESD for EGC-MH. EGC-MH is associated with more submucosal invasion and higher risk of LNM or LVI than pure-type gastric cancer, irrespective of whether the mixed component is differentiated or undifferentiated, although the mechanism is unclear.^[7,20–25]^ In terms of the therapeutic outcomes, retrospective analysis of surgical data showed that EGC-MH showed no LNM among lesions that met the present ESD criteria.^[26]^ Retrospective analysis of ESD data also showed that differentiated-type predominant EGC mixed with an undifferentiated component showed no LNM or extragastric recurrence if the lesions met the present ESD criteria.^[27]^ However, another retrospective analysis of endoscopic resection data showed that differentiated-type predominant EGC mixed with an undifferentiated component was a significant risk factor for noncurative resection regardless of tumor size.^[28]^ The reason of performing this systematic review and meta-analysis is this heterogeneity among the publications about therapeutic outcomes of EGC-MH. Simple discrimination into differentiated-type predominant, or undifferentiated-type predominant mixed EGC might not reflect the feasibility of ESD for EGC-MH.

Mixture of poorly differentiated adenocarcinoma (PDC) and signet ring cell carcinoma (SRC) is another issue. Both of these cancers are included in the EGC-UH in the Korean or Japanese guidelines; however, there have been no considerations for the treatment for mixture of PDC and SRC. In a retrospective analysis of surgical data, a mixture of PDC and SRC showed an LNM rate of 6.3%, which is higher than in pure-type PDC or SRC.^[29]^ Moreover, an LNM rate of 5.3% was observed when this mixture of PDC and SRC met the current ESD criteria.^[29]^ Retrospective analysis of ESD data also showed that a mixture of PDC and SRC showed lower curative resection rates than pure SRC (77.7 vs 93.8%) and was associated with noncurative resection in a multivariate analysis, in addition to more submucosal invasion and positive vertical margins.^[30]^

The results of this study will provide evidence for validity of current ESD criteria in addition to the technical feasibility of ESD for EGC-MH.

## Author contributions

**Conceptualization:** Chang Seok Bang.

**Data curation:** Chang Seok Bang, Jae Ho Choi, Young Joo Yang, Jae Jun Lee, Gwang Ho Baik.

**Formal analysis:** Chang Seok Bang, Jae Ho Choi.

**Funding acquisition:** Chang Seok Bang.

**Investigation:** Chang Seok Bang, Jae Ho Choi, Young Joo Yang, Jae Jun Lee, Gwang Ho Baik.

**Methodology:** Chang Seok Bang.

**Project administration:** Chang Seok Bang.

**Resources:** Chang Seok Bang, Jae Ho Choi, Young Joo Yang, Jae Jun Lee, Gwang Ho Baik.

**Visualization:** Chang Seok Bang.

**Writing – original draft:** Chang Seok Bang.

**Writing – review & editing:** Chang Seok Bang.

Chang Seok Bang orcid: 0000-0003-4908-5431.
